# Effect of Hexagonal Boron nitride Nanopowder Reinforcement and Mixing Methods on Physical and Mechanical Properties of Self-Cured PMMA for Dental Applications

**DOI:** 10.3390/ma13102323

**Published:** 2020-05-19

**Authors:** Mana Alqahtani

**Affiliations:** Department of Surgery, University of Tabuk, Tabuk 71491, Saudi Arabia; maalqahtani@ut.edu.sa

**Keywords:** self-cured PMMA, BN nanoparticles, structural properties, mechanical properties

## Abstract

We report for the first time on the effect of biocompatible hexagonal boron nitride (h-BN) nanopowder reinforcement with different concentrations on the structural and mechanical properties of fabricated self-cured polymethyl methacrylate (PMMA) based dental materials (GC UNIFAST III). A comparison among the structural and mechanical properties between hand and ultrasonic mixing is also presented. Fabricated specimens were studied by scanning electron microscopy (SEM), X-ray diffraction (XRD), Fourier transform infrared spectrometry (FTIR), micro indentation, and flexural strength techniques. The ultrasonic mixing method provides better sample textures of the composite as compared to hand mixing. It is found that XRD and IR intensity of the peaks increases with the increase of h-BN concentration due to nanocomposite formation. The additions of h-BN nanoparticles to the acrylic resin enhanced the hardness and the flexibility values of the composites. Independently of the mixing method used, adding h-BN nanopowder relatively increases the Vickers Hardness numbers (VH) and Flexural Strength (FS) of the unmodified materials. However, using ultrasonic mixing method combined with h-BN nanopowder increases VH numbers to 300% and FS values to 550% with respect to the unmodified sample made by hand mixing. The results obtained are very encouraging and will support future research in vivo, to confirm whether PMMA loaded with h-BN nanoparticles is an improvement compared to current dental restorative materials.

## 1. Introduction

Polymethyl methacrylate (PMMA) is the preferred resin material used in restorative dentistry because of its low water sorption and solubility, lack of toxicity, simple manipulation technique, excellent aesthetics, and reparability. Non-modified PMMA tends to be brittle when subjected to an impact force. The hand mixing method of self-cured PMMA material being currently used in dental offices should be performed in less than 20 s as the dough hardens very quickly into its final configuration. In doing so, the mechanical properties of the composite are not satisfactory and often result in brittle materials used in dental repairs for instance [1,2,3]. Studies have focused on the development of curing methods, resin monomer [4], and the pre-treatment of inorganic fillings [5,6] with the objective to improve the characteristics of resin-based compounds. Heat treatment and post treatment increase the degree of polymerization and, to some extent, improves the strength of the composite [7]. In addition, several studies have attempted to improve the impact strength, fatigue resistance and transverse strength of the acrylic resin. Reinforcing with carbon fibers, glass fibers, ultra-high molecular, and metal powders are one of the modifications to produce an acrylic co-polymerized with relatively high impact strength [8,9,10]. A systematic review and meta-analysis was also carried out to examine the mechanical properties of provisional restoration materials used with direct techniques [11]. 

The use of advanced nanomaterials is currently paving the future of dentistry by improving the health care of dental patients. Efforts are aimed at using nanoparticles in order to reinforce the acrylic resins, therefore producing a polymer nanocomposite with improved physical and mechanical properties as compared to those filled with microparticles. However, the mechanical mixing of constituents tends to be ineffective and often result in poor filler dispersion in the polymer matrix, especially when the filler size is in the nanometer range. Furthermore, dental applications require high-quality materials that are biocompatible, wear-resistant, and durable. Zirconia and emerging h-boron nitride (BN) materials perfectly meet these conditions. In this work, we are focusing on biocompatible fillers and metal-free that improve the mechanical performance of the dental materials. Other materials such as graphene, metals, and other ceramics may be used as fillers in PMMA for non-dental purposes such as plasticizer in bone replacement cements, acrylic glass, etc. Within the field of dentistry, zirconia is considered one of the biocompatible fillers that is being tested to improve the fracture resistance of dentals materials. The addition of zirconia to the glass ionomer cement (GIC) which is branded as Zirconomer^®^ has improved the mechanical properties compared with Amalgam. The GIC has a compressive strength significantly lower compared with Zirconomer^®^, 107 MPa versus 195 MPa [12,13].

The hexagonal boron nitride (h-BN) as a fine-grained lubricant is used in paints, cosmetics, and dental cements [14]. It is also currently used by nearly all leading manufacturers of cosmetic products including make-up accessories and other skincare products [15]. A solution mixing method was used to prepare PMMA based composites blended with h-BN [16,17]. An electrostatic nano-assembly method was also carried on to make h-BN/PMMA in order to promote the heat-conducting properties of the composite [18]. Moreover, PMMA composites were reinforced with boron nitride nanotubes (BNNT) by using in-situ polymerization process to enhance the thermal conductivity of the nanocomposite [19]. Using boron powder and metal oxide as reactants under chemical vapor deposition method and a 1wt.% BNNTs fraction in PMMA, the elastic modulus of the composite was increased up to 19% [20]. The best results have been obtained so far when using amine functionalized BN nanoflakes which were entrained in PMMA sheet to improve the mechanical properties of the composite [21]. In this work, modulus of elasticity and strength were increased by 148% and 155%, respectively, at BN concentration of 2% per weight. 

A developed nano-size h-BN was reported to prevent the formation of dental calculus [22]. Boron nitride nanotubes were also used as novel fillers for improving the properties of dental adhesives [23]. In general, the BN hardness is lower than diamond. As a result of its excellent chemical and thermal stability, BN is widely used in mechanical applications. The properties of the reinforced material depend on the size, shape, type, and concentration of the added particles to the host monomers [24].

To our knowledge, we report for the first time on comparison among hand and ultrasonic mixing of boron nitride reinforced PMMA for dental restorations. We also examined the effect of nano-sized hexagonal boron nitride (h-BN) powder reinforcement with different concentrations on the structural, hardness, and flexural strength properties of fabricated specimens. These are investigated by using scanning electron microscopy (SEM), X-ray diffraction (XRD), Fourier transform infrared spectrometry (FTIR), micro indentation, and flexural strength measurement techniques. This work is presented as follows: Section 2 shows details on the experimental process. The results and discussion are given in Section 3, while Section 4 summarizes the conclusions.

## 2. Materials and Methods 

### 2.1. Specimens Preparation

The preparation of the specimens was made by mixing the resin UNIFAST III powder with UNIFAST liquid monomer from GC Corporation (Tokyo, Japan). The standard powder to liquid ratio was 1g of powder to 0.5 mL of liquid (1 scale of powder to 1 scale of liquid), however the powder to liquid ratio is adjustable between 2 g:1 mL and 2 g:1.5 mL. Both hand and ultrasonic mixing were carried out in this study. The hand mixing was made exactly as routinely performed at a dental office, where powder and liquid monomer are put together in a rubber cup and hand mixed for 15–20 seconds using a plastic spatula. The ultrasonic mixing is carried out by using high power Ultrasonic Homogenizer Sonicator Cell Disruptor Mixer 450 W 10–300 mL (U.S. Solid, Cleveland, OH, USA). The power of the ultrasonic homogenizer was kept between 5% and 50% of maximum power of 450 Watts for 20 s of mixing time. When the mixture reaches a soft dough stage, it was immediately poured into 15 mm diameter by 3 mm thick disc shape Teflon molds. The mold which provides extremely high tear resistance has both ends opened, so a compression molding technique consisted of pressing specimens sandwiched between two plates made of glass. The discs are then released after the short thermal setting of the composites while extra material was removed using a razor blade between each compression. For the BN reinforcement fillers, 99.8% purity h-BN nanoparticles, 50–70 nm and 800 nm in size, were purchased from US Research Nanomaterials Co., Ltd. (Houston, TX, USA) and used in our experiments. The proper loading of BN nanofillers for each mixture were measured using an available Uuni-WT 100 × 0.1 micro gram Analytical Balance Lab with Digital Scale Range. We made typical mixtures of 0.5, 1.0, 3.0, and 5.0% by weight (wt.) of loading nanoparticles into UNIFAST III powder-based solution. Nanoparticles easily clump due to high surface energy and many conventional techniques cannot break them apart, therefore making well distributed nanoparticles in polymer films is the key issue to achieving higher mechanical performance. Prior to mixing BN nanoparticles with the powder, a solution of h-BN nanoparticles in GC UNIFAST liquid solvent was sonicated to completely disperse the nanoparticles. We also made unloaded or control samples for comparison purpose.

### 2.2. Characterization

The surface morphology of prepared samples was recorded using a scanning electron microscope TESCAN VEGA (Model 3 LMU, Bangkok, Thailand). Before recording the images, the samples were coated with a thin layer of gold to make them electrically conductive. The XRD patterns of the samples were recorded using X-ray diffractometer (Model X’PERT-3, Kassel, Germany), powder using CuKα radiation (1.5 Å) with continuous scan from 10–80 degree with scan rate of 0.03/s. FTIR spectra were recorded using Perkin-Elmer Spectrometer (Model S-600, Llantrisant, UK) in the KBr medium. The samples were scanned in the wavelength range 400–4000 cm^−1^. The micro-hardness characterization of the samples was investigated by Vickers hardness measurement technique using Vickers hardness tester (Model: VH-5B Kolkata, India). The specimen is prepared as per American Society for Testing of Materials (ASTM) norm, then it is polished in a polishing machine using rough polishing and fine polishing using a 3 micron grade polishing paste, after getting micro polishing specimen is tested by a hardness testing machine to determine the Vickers micro hardness number by using a diamond indenter of few microns diameter. The indenter is made to strike the specimen samples for varying loadings. In each measurement the indenter is allowed to strike the sample for a period of 20 s then the average indentation diameter (average of both diagonals of the indenter) of the sample is recorded using the travelling microscope attached to the testing machine. The Vickers hardness number in Kg/mm^2^ is calculated using the formula:

(1) $$HV=\left(\frac{1.854f}{d^{2}}\right)$$

where f is the applied force in kilogram-force (Kgf) and d is the diagonal indentation length in micrometer. To convert HV to MPa we multiplied the recorded values by 9.807. The Vickers hardness (VH) measurements on specimens made by hand and ultrasonic mixing methods include the control sample and nano-sized h-BN reinforced samples with different concentrations. 

After manufacturing, each group of specimens were cut out of blocks with a diamond saw to enable measurements of the flexural strength. All bending bars (3 × 2.5 mm) were polished using diamond-embedded wheels of 45 µm grit size with water running and then to 2.5 and 1 µm with polycrystalline diamond paste. The flexural strength of the specimens was evaluated via the three-point bending test (Instron Model 3355, Bangalore, India) with an across head speed of 0.4 mm/min. The load and the corresponding deflection were recorded. The flexural strength was calculated using the following formula:

(2) $$\delta_{f}=\left(\frac{3fl}{2wh^{2}}\right)$$

where f = load at fracture, l = span tested (here 8mm), w = width of the specimen, h = height of the specimen.

## 3. Results and Discussion

**Figure 1:**
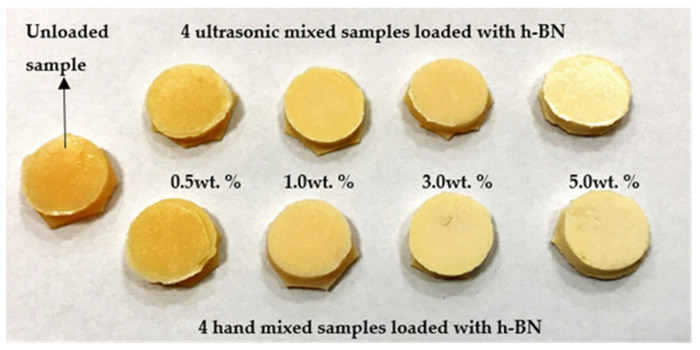
Pictures of a typical control sample and specimens made by mixing nano-sized hexagonal boron nitride (h-BN) powder reinforcement with different concentrations into PMMA materials.

Figure 1 shows the pictures of a typical control sample and specimens made by mixing nano-sized h-BN powder reinforcement with different concentrations into PMMA materials. 

**Figure 2:**
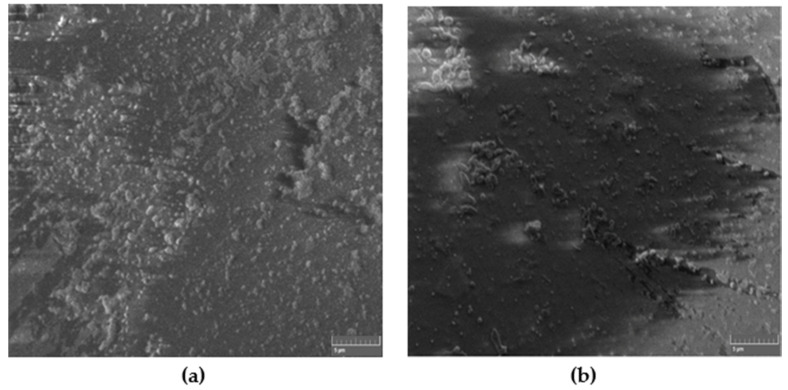
SEM images of non-modified self-cured composite for (**a**) hand and (**b**) ultrasonic mixing methods.

Figure 2 shows the SEM images of a typical non-modified self-cured composite for both hand and ultrasonic mixing. Apparently, and for the same magnification, the ultrasonic mixing method provides better textures and packing density of the composite as compared to hand mixing.

**Figure 3:**
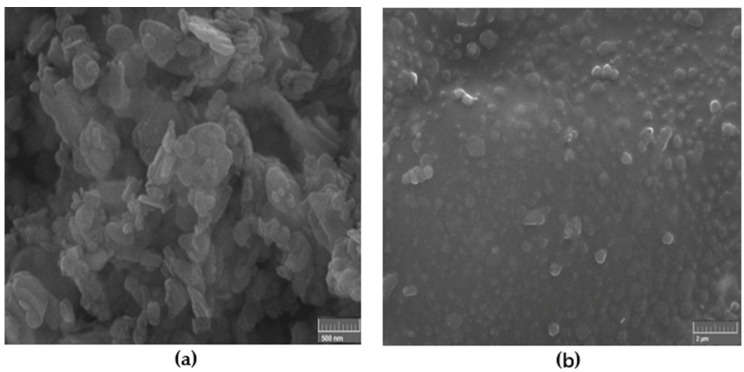
SEM image of: (**a**) high-resolution h-BN nanoparticles 50–70 nm which were used as reinforcement fillers and (**b**) nanocomposite reinforced with 0.5 wt.% of such nanoparticles under ultrasonic mixing.

Figure 3 shows the SEM images of the high-resolution h-BN nanoparticles 50–70 nm which were used as reinforcement fillers and the nanocomposite reinforced with 0.5 wt.% of such nanoparticles under ultrasonic mixing. The fillers are expected to modify the structural and mechanical properties of the nanocomposites. 

**Figure 4:**
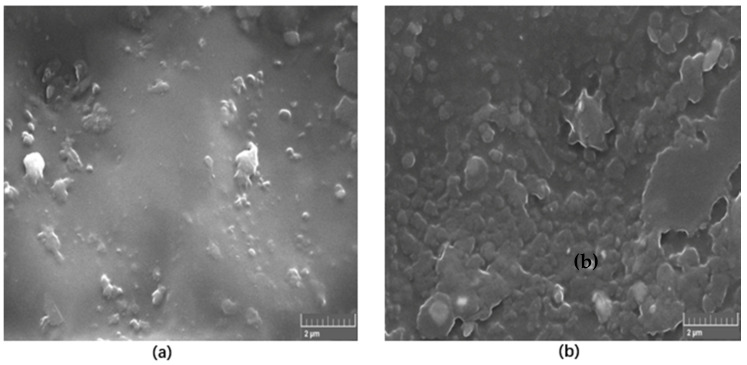
SEM images of (**a**) 1 wt.% and (**b**) 5 wt.% self-cured PMMA/h-BN nanocomposites formed under ultrasonic mixing.

Figure 4 shows a typical SEM images of 1 wt.% and 5 wt.% self-cured PMMA/h-BN nanocomposites formed under ultrasonic mixing. It is found that the nanoparticles are highly dispersed, and the texture is significant as we increase the amount of h-BN material into the composite.

**Figure 5:**
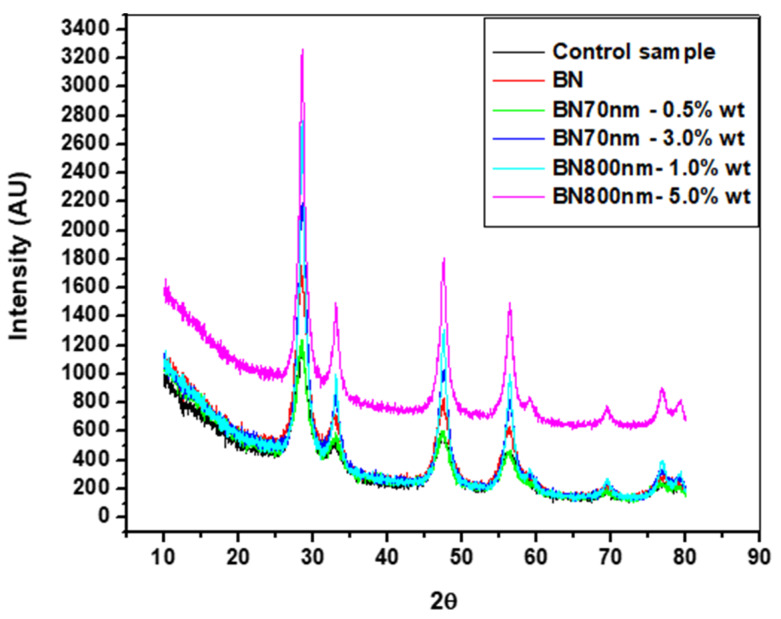
XRD patterns of the specimens made by ultrasonic mixing with different h-BN nanofillers in PMMA.

Figure 5 shows the XRD patterns of the specimens made by ultrasonic mixing and which include starting materials and nano-sized h-BN reinforcement with different concentrations. In the XRD observations four strongest peaks are detected at Bragg angles 27.5°, 33°, 47°, and 57°. The peaks with Miller indices (002), (100), and (102) at 27.5°, 47°, and 57°, respectively, correspond to pure crystalline h-BN nanoparticle fillers. The control sample which represents bare PMMA has a semi-crystalline nature with a primary pick at 27.5°. It is found that intensity of the peaks increases with the increase of h-BN concentration due to nanocomposite formation. This is true for 0.5 wt.% versus 3.0 wt.% for 70 nm h-BN fillers as well as 1.0 wt.% versus 5.0 wt.% for 800 nm h-BN fillers in PMMA. The peak corresponding to 1.0 wt.% PMMA/BN800 shows higher intensity than the peak corresponding to 3.0 wt.% PMMA/BN70 nm because of the large size of h-BN nanoparticles. The primary peaks exhibit higher intensity reflecting the good crystalline nature of the nanocomposites. The final product is a pure PMMA mixed with h-BN nanopowder as no peaks corresponding to impurities are detected.

**Figure 6:**
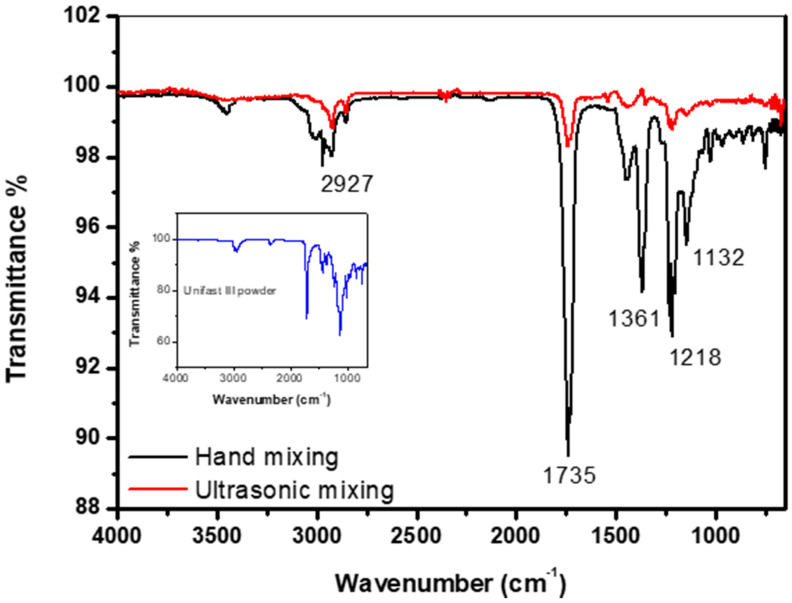
FTIR spectrum of control specimen made by hand and ultrasonic mixing methods as well as the spectrum corresponding to UNIFAST III powder.

Figure 6 shows the FTIR spectrum of the control specimens made by both hand and ultrasonic mixing methods as well the spectrum corresponding to GC UNIFAST III powder. The band at around 1132 cm^−1^ is the characteristic absorption vibration of PMMA [25]. The bands at about 1218 cm^−1^, 1361 cm^−1^, 1735 cm^−1^, and 2927 cm^−1^ are assigned to υ(C-O) stretching vibration, wagging vibration of C–H, C=O stretching, and C-H stretching, respectively. It is also found that specimens made by hand and ultrasonic mixing do not show similar FTIR spectrum. The spectrum of the specimen made by hand mixing is much similar to the one taken on pure GC UNIFAST III powder (Figure 6 inset). This could be a sign that polymerization of the powder under liquid monomer is not fully complete as compared to specimen made by ultrasonic mixing.

**Figure 7:**
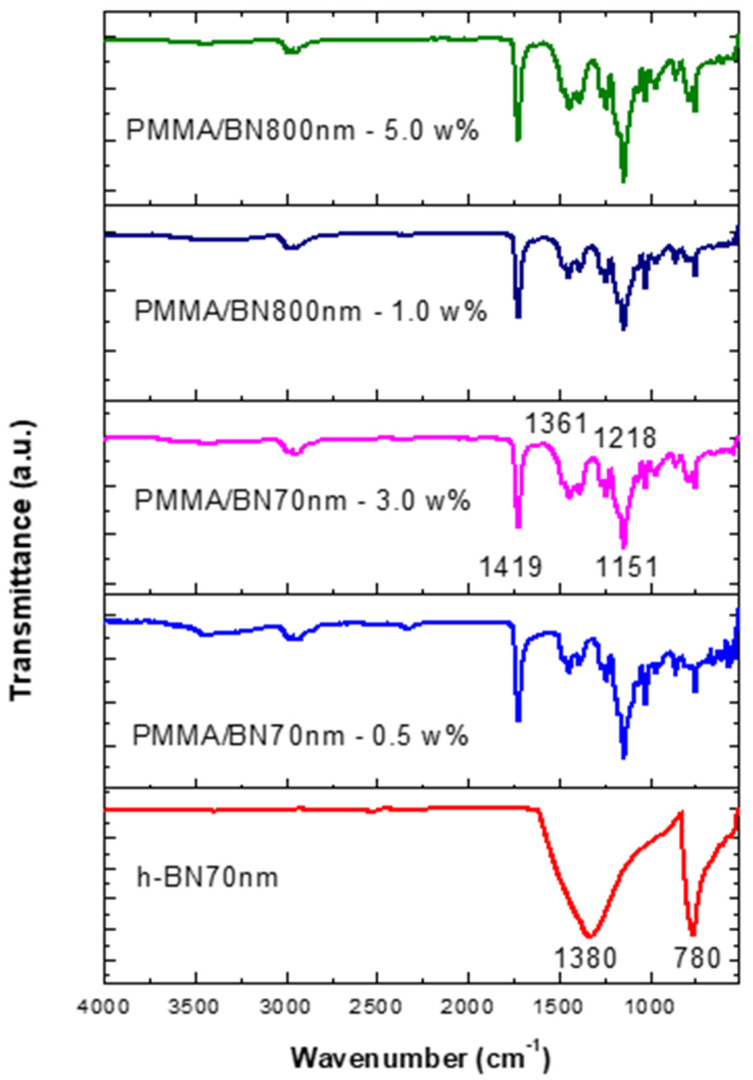
FTIR data of specimens made by ultrasonic mixing for nano-sized h-BN reinforcement with different concentrations.

Figure 7 shows the FTIR data of the specimens made by ultrasonic mixing and which include starting materials and nano-sized h-BN reinforcement with different concentrations. Since Geick [26] published IR characteristics of h-BN, several researchers have given IR data on a variety of thin BN films [27,28,29,30,31,32,33,34]. Most investigators agree that there are two distinct IR absorption bands in boron nitride films. These are the band around 1380 cm^−1^ (in plane) and the band around 780 cm^−1^ (out of plane) which is due to B-N stretching and B-N-B bending, respectively. Entraining h-BN nano-powder into PMMA resulted in a modification of the molecular structure and therefore a change in the absorption spectrum. The local bands of the control samples at 1218 cm^−1^, 1361 cm^−1^ have shifted to 1151 cm^−1^, 1419 cm^−1^ as a result of PMMA interaction with the h-BN nanoparticles active mode at 1380 cm^−1^. The intensity is shown to increase with the increase of h-BN for a respective size. 

**Figure 8:**
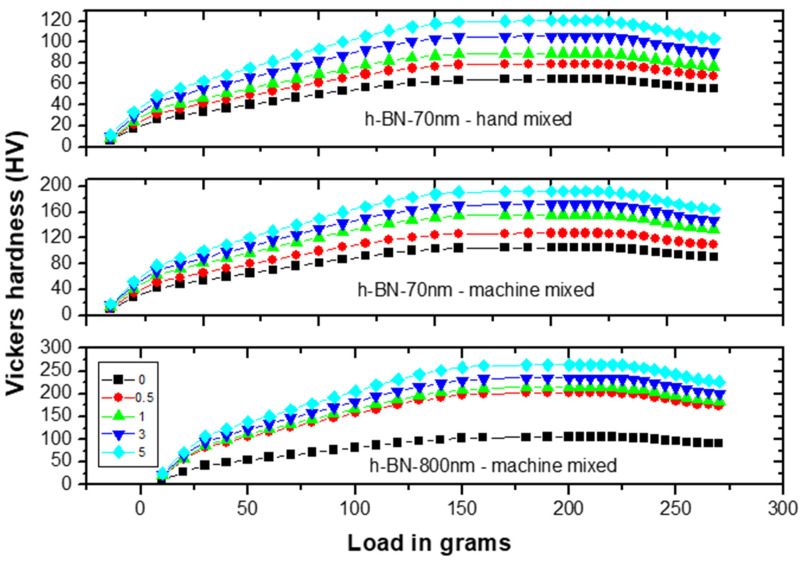
Vickers hardness (VH) measurements versus load on specimens made by hand and ultrasonic mixing methods for 70 nm and 800 nm h-BN reinforcement with different concentrations.

Figure 8 shows the Vickers hardness (VH) measurements on specimens made by hand and ultrasonic mixing methods and which include the control sample and 70 nm and 800 nm nano-sized h-BN reinforced samples with different concentrations. Initially, VH numbers increase with increasing load indicating the material resistance to plastic deformation. Then, VH stabilizes around a certain value (plateau region) at a load of 160 gf which defines the hardness value of the material beyond which the mechanical breakdown of the materials takes place at a load of 225 gf. It is found that recorded VH hardness are 1019 MPa versus 617 MPa for control specimens prepared by ultrasonic and hand mixing methods, respectively. This is translated to a more than 65% increase in the hardness value when using ultrasonic mixing (Figure 8). For specimens loaded with nano-sized h-BN powder, VH numbers increases with the increasing of h-BN fillers concentration. For specimens prepared by ultrasonic mixing, VH hardness values reached 1863 MPa when using h-BN 70 nm and 2569 MPa when using h-BN 800 nm in size at the same concentration of 5.0 wt.%. 

**Figure 9:**
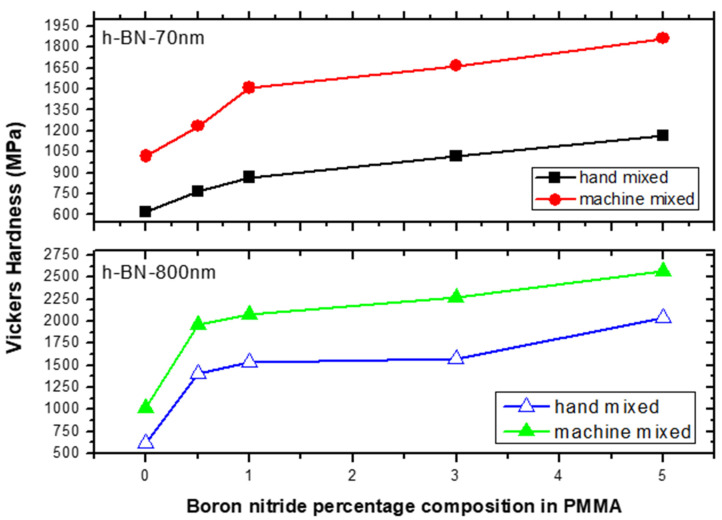
Vickers hardness (VH) measurements versus h-BN composition in PMMA on specimens made by hand and ultrasonic mixing methods.

Figure 9 depicts the VH hardness measurements versus h-BN composition in PMMA on specimens made by both hand and ultrasonic mixing methods. The h-BN 70 nm are less effective than h-BN 800 nm with a relative difference in hardness of about 40%. We notice that even at lower filler concentration of 0.5 wt.%, the hardness value double and triple when using h-BN 70 nm and 800 nm in size, respectively. It shows that using ultrasonic mixing method combined with 5.0 wt.% of nano-sized h-BN powder, increases the VH hardness to more than 300% with respect to the unmodified sample made by hand mixing.

**Figure 10:**
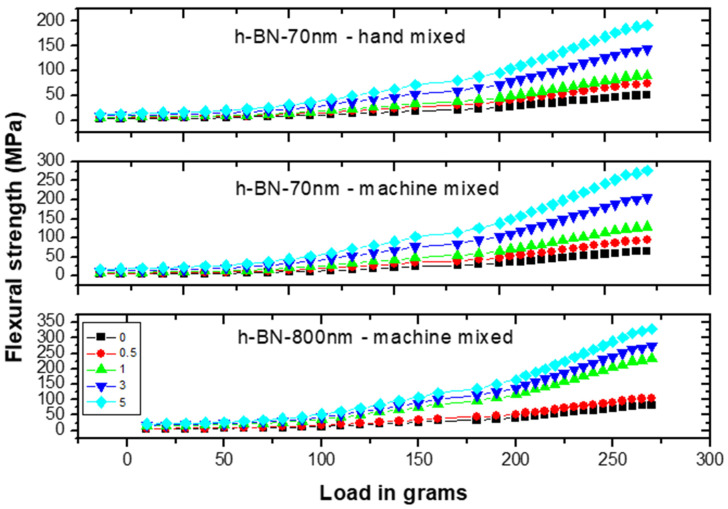
Flexural strength (FS) measurements versus load on specimens made by hand and ultrasonic mixing methods for 70 nm and 800 nm h-BN reinforcement with different concentrations.

Figure 10 shows the flexural strength (FS) measurements versus load on specimens made by hand and ultrasonic mixing methods for 70 nm and 800 nm h-BN reinforcement with different concentration. FS initially increases with increasing load indicating the material degree of rigidity. Recorded stiffness strength values were 50 MPa versus 65 MPa for control specimens prepared by ultrasonic and hand mixing methods, respectively.

**Figure 11:**
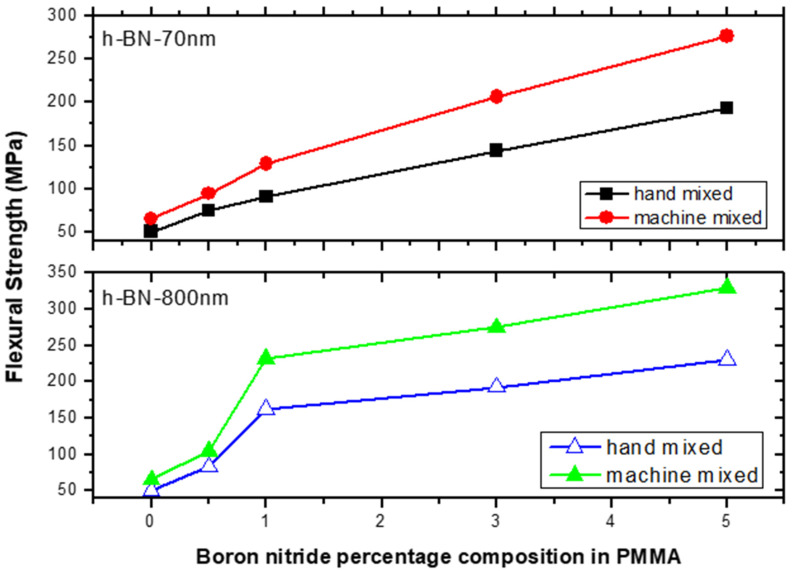
Flexural strength (FS) measurements versus h-BN composition in PMMA on specimens made by hand and ultrasonic mixing methods.

Figure 11 depicts the flexural strength measurements versus h-BN composition in PMMA on specimens made by both hand and ultrasonic mixing methods. It was about 330 MPa when using h-BN 800 nm in size at a concentration of 5.0 wt.%. It shows that using ultrasonic mixing method combined with nano-sized h-BN powder increases the FS values to more than 550% with respect to the unmodified sample made by hand mixing. At lower filler concentration of 0.5 wt.%, the flexural strength value double with h-BN 800 nm being more effective in enhancing the flexural strength.

In fact, any significant increase in the hardness and/or bending stress would be beneficial to dental materials and provisional restorations’ deficiencies. A similar behavior is observed when halloysite nanotubes (HNTs), an aluminosilicate clay mineral, was incorporated in PMMA. HNTs were shown to increase flexural strength and surface microhardness of the composite [35]. A small amount (0.3% per weight) of HNTs significantly improved the hardness values but not the flexural strength of PMMA. In contrast to h-BN nanopowder, addition of 0.6 or 0.9 wt.% of HNTs resulted in flexural strength and hardness lower than those of the control specimens. As the size of HNTs are hundreds of nanometers in length and tens of nanometers in diameter, they probably tend to form clusters (structural defects) that develop mechanical weak points. Similar results were also reported when adding 0.5–5.0 wt.% of aluminum oxide powder to PMMA. Addition of 2.5 wt.% aluminum oxide powder to PMMA significantly increased the hardness and flexural strength of a conventional heat-cured acrylic resin [36]. In our case, although a minimum filler concentration (0.5% per weight) will increase the mechanical performance of PMMA composites, we believe that using a higher amount of biocompatible h-BN fillers (5.0 wt.%) will not affect the color stability of the dental composite because of the natural white color of the h-BN nanofiller. 

## 4. Conclusions

Specimens made of different size boron nitride nanopowder-reinforced self-cured PMMA materials were fabricated by using both hand and ultrasonic mixing methods and then investigated with respect to their structural and mechanical properties. Within the limitations of this study, the following results were obtained: ultrasonic mixing method provides better textures and packing as compared to hand mixing. As expected, h-BN fillers modified both structural and hardness properties of the composites as evidenced by XRD, FTIR, and micro indentations measurements. It is found that the nanoparticles are highly dispersed, and the texture is significant as we increase the amount of h-BN material into the composite. The XRD intensity of the peaks increases with the increase of h-BN concentration due to nanocomposite formation. However, because of the h-BN nanoparticles larger size, the peak intensity is relatively higher. The final product was a pure PMMA mixed with h-BN nanopowder as no peaks corresponding to impurities were detected. It is also found that specimens made by hand and ultrasonic mixing do not show similar FTIR spectrum. In hand mixing, polymerization of the powder under liquid monomer is not fully complete as demonstrated by specimen made by ultrasonic mixing. Inclusion of h-BN nano-powder into PMMA resulted in a change of the absorption spectrum. The IR peak intensity is shown to increase with the increase in size of h-BN nanopowder. As expected, additions of h-BN nanoparticles to PMMA enhanced the hardness and flexural strength values of PMMA. Independently of the mixing method used, adding nano-sized h-BN powder, relatively increases the VH numbers and FS values of the unmodified materials. However, using ultrasonic mixing method combined with nano-sized h-BN powder increases VH numbers to 300% and FS values to 550% with respect to the unmodified sample made by hand mixing. The results obtained are very encouraging and will support future research in vivo, to confirm whether PMMA loaded with h-BN nanoparticles is an improvement compared to current dental restorative materials.
